# Colon capsule endoscopy today: Brief overview of leading UK and Danish initiatives

**DOI:** 10.1055/a-2641-5952

**Published:** 2025-07-23

**Authors:** Jakob Frederik Frokjaer Justsen, Niels Gellert Olesen, Gunnar Baatrup, Anastasios Koulaouzidis

**Affiliations:** 1 11286 Kirurgisk Afdeling A, OUH, Odense, Denmark; 2 74340 Department of Surgery, Odense University Hospital,, Syddansk Universitet Det Sundhedsvidenskabelige Fakultet, Odense, Denmark; 3 6174 Department of Clinical Research, SDU, Odense, Denmark; 4 11286 Afdeling A, OUH, Odense, Denmark; 5 37805 Department of Gastroenterology, Pomeranian Medical University in Szczecin, Szczecin, Poland

**Keywords:** Endoscopy Small Bowel, Capsule endoscopy, Endoscopy Lower GI Tract, CRC screening, Colorectal cancer, Polyps / adenomas / ...

## Abstract

**Background and study aims:**

In recent years, several large national studies have been published reporting on outcomes of colon capsule endoscopy (CCE) in both symptomatic and screening settings, significantly contributing to the expanding body of real-world evidence on CCE. Therefore, we have compiled these studies to provide an overview of key developments, current challenges, and valuable insights they offer into the evolving role of CCE.

**Patients and methods:**

We examined three multicenter studies reporting on outcomes of CCE including the NHS England study with 4,878 symptomatic patients; the ScotCap pilot with 316 symptomatic patients; the ScotCap registry with 1,087 predominantly symptomatic patients (95.9%); and the CareForColon 2015 study with 1,790 patients in a screening setting. For the ScotCap pilot study, only symptomatic patients were included.

**Results:**

ScotCap pilot reported the highest rate of adequate bowel preparation (79.4%) without using prucalopride. CareForColon2015 achieved a significantly higher rate of complete tests (91.7%) compared with other studies. NHS England reported a notably lower rate of follow-up endoscopy (46.7%), indicating effective patient selection. ScotCap pilot reported one case of missed colorectal cancer. Sensitivity of CCE for detecting polyps ≥ 10 mm ranged from 93.8% to 97.0% on a per-patient basis and from 75.0% to 95.8% on a per-polyp basis in the NHS England and ScotCap trials.

**Conclusions:**

These national CCE programs reveal the complexity of large-scale implementation, driven by variations in definitions and protocols. Harmonized quality metrics and shared definitions of success are essential. Efforts should focus on reducing downstream procedures and fostering cross-system learning.

## Introduction

In recent years, several large national studies have been published reporting on outcomes of colon capsule endoscopy (CCE) in both symptomatic and screening settings, significantly contributing to the expanding body of real-world evidence on CCE. Notably, the recent study by Tuvill et al. examined use of CCE in symptomatic patients across NHS England 1 , while the Danish CareForColon2015 trial 2 assessed performance of CCE within a national fecal immunochemical test (FIT)-positive screening cohort. These findings are further complemented by data from the ScotCap pilot and registry studies conducted in Scotland 3 4 . Together these national initiatives represent the most comprehensive real-world datasets on CCE to date.

To illustrate key developments and challenges in CCE, we compiled and tabulated comparative national-level data from England, Scotland, and Denmark, highlighting variability in clinical settings, bowel preparation regimens, outcome definitions, and overall performance ( Table 1 ). Although cross-comparisons of this kind are inherently limited by differences in referral patterns, reading experience, patient preparation protocols, and definitions of completeness and adequate bowel preparation, they nonetheless offer valuable insights into the scalability of CCE.

**Table:** **Table 1** Study results.

Study	Setting	N (CCE)	Complete test (%)	Adequate bowel prep (%)	Successful test (%)*	No further test (%)	FUE (%) **^†^**	CRC (%)	Prucalo-pride	Missed CRC ^§^
NHS England 2025 1	Symptomatic (England)	4,878	74.0	74.0	63.0	50.0	46.7	1.1	Yes (35/55 centers)	0
ScotCap pilot 2021 3	Symptomatic (Scotland)	316	72.2	79.4	65.8	37.3	58.2	0.0	No	1
ScotCap registry 2024 4	Mixed (95.9% symptomatic) (Scotland)	1,087	57.0	59.2	56.9	42.0	56.8	1.2	No	0
CareForColon2015 2025 2	Screening (Denmark)	1,790	91.7	75.9	69.2	30.1	69.9	3.5 ^‡^	Yes	–
* Defined as a complete CCE with adequate bowel preparation. † Follow-up endoscopy (colonoscopy or flexible sigmoidoscopy). ‡ Overall percentage for the intervention arm, CCE accounted for only 32.6% of the procedures. §Incomplete and adequate prepared tests.										

## Bowel preparation

In CCE, adequate bowel cleanliness is crucial, because suboptimal visualization of colonic mucosa increases cancer and polyp miss rates 5 . The European Society of Gastrointestinal Endoscopy (ESGE) sets a 90% adequacy rate for bowel preparation in colonoscopy with a target of ≥ 95% 6 . Despite observed improvements, none of the studies reached this benchmark. The highest reported rate of adequate bowel preparation (79.4%) was in the ScotCap pilot, which used the Boston Bowel Preparation Scale, defining adequate as ≥ “fair” in all colonic segments 3 . Conversely, the ScotCap registry reported the lowest rate (59.2%) using the Leighton–Rex Bowel Preparation Scale with the same definition of adequate 4 . Both ScotCap studies employed the same bowel preparation regimen, suggesting that differences may be attributable to population characteristics or scoring criteria. NHS England and CareForColon2015 reported moderate adequacy rates (74.0% and 75.9%, respectively), defining adequate as a score ≥ 6 on the Colon Capsule Clear Score (CC-Clear) and a score ≥ fair on the Leighton–Rex scale for all visualized colonic segments, respectively 1 2 . Definitions of adequate bowel preparation used in the studies are summarized in Supplementary Table 1.

Regimens for bowel preparation differed among the studies ( Table 2 ). One important difference between studies was use of prucalopride (Resolor), a 5-HT4 receptor agonist that enhances colonic transit by increasing peristaltic reflex and colonic contractions 7 . Adding prucalopride to the bowel preparation regimen has been shown to increase both completion rates and the proportion of acceptable bowel preparations in CCE 8 . Prucalopride was included in bowel preparation of both NHS England and CareForColon2015, which also reported the highest rates of complete test (74.0% and 91.7%, respectively) 1 2 . In NHS England, however, prucalopride was only used in 35 of 55 centers, and some centers lacked access to gastrografin boosters, which may have adversely affected rates of complete test and adequate bowel preparation. In contrast, prucalopride was not used in either of the ScotCap studies, possibly contributing to the lower adequacy and completion rates observed, particularly in the registry 4 . Interestingly, the ScotCap pilot achieved complete test rates comparable to those in NHS England and reported the highest percentage of adequate bowel preparation among the studies, highlighting the importance of factors beyond pharmacological agents, such as patient population and test quality criteria, in influencing outcomes 3 .

**Table:** **Table 2** Bowel preparation protocols in the studies.

NHS England 1	3-day low-residue diet, with 2 split doses of polyethylene glycol-electrolyte taken the evening before and morning of procedure Two boosters comprising gastrografin and phosphosoda after ingestion of CCE. If needed bisacodyl suppository at the end of the day (some centers only had access to phosphosoda) 35/55 centers used prucalopride
ScotCap registry 4	3 days before procedure: 1 sachet of Macrogol 3350 in 125 mL water morning and evening 2 days before procedure: 1 sachet of Macrogol 3350 in 125 mL water morning and evening 1 day before procedure: 2 L polyethylene glycol taken over 2 h in the evening Day of procedure: 2 L polyethylene glycol taken over 2 h in the morning, 10 mg metoclopramide hydrochloride tablet after ingestion of CCE, 1 L of sodium picosulfate taken as indicated by the three data recorder signals and 1 bisacodyl suppository (optional) if CCE not excreted 10 h after ingestion
ScotCap pilot 3	Same as the ScotCap registry, but on the procedure day, if bowel cleanliness was inadequate, Picolax in 1 L of water was administered
CareForColon2015 8	3 days before procedure: 2x polyethylene glycol 13,8 g sachet, normal diet, 2 L water 2 days before procedure: 2x polyethylene glycol 13,8 g sachet, normal diet, 2 L water 1 day before procedure: 1 L polyethylene glycol incl. ascorbic acid + 1 L water, clear liquid diet Day of procedure before capsule intake: 1 L polyethylene glycol incl. ascorbic acid + 1 L water, clear liquid diet, 2 mg prucalopride tablet 45–60 min before ingestion of CCE Day of procedure after capsule intake: chewing gum, signal 1: 330 mL sulfate-based solution + 2–3 large glasses of water, signal 2: 330 mL sulfate-based solution + 2–3 large glasses of water, signal 3: 330 mL sulfate-based solution + 2–3 large glasses of water, 200 mg caffeine tablet and a small fatty snack, signal 4: 10 mg suppository bisacodyl

As of this writing, the current ESGE guidelines for bowel preparation in CCE have not been updated since 2012 9 . Together, these findings suggest that further optimization of bowel preparation regimens, including more consistent use of prokinetic agents, remains a priority for improving CCE performance.

## Complete test

Complete colonic visualization is another cornerstone for successful CCE examination. CareForColon2015 achieved a significantly higher rate of complete tests (91.7%) compared with the other studies, defining a complete test as visualization of the hemorrhoidal plexus 2 10 . In comparison, NHS England (74.0%) and the ScotCap pilot (72.2%) had lower rates, with NHS England defining a complete test as the capsule being seen expelled or identification of anal cushions, and the ScotCap pilot defining it as excretion of the capsule within its battery life or visualization of the anal cushions 1 3 . The ScotCap registry reported the lowest rate of complete tests (57.0%), with a complete test defined as visualization of the entire colon and rectum 4 . Notably, The ScotCap registry delivered services in a more decentralized manner, including community-based sites – an approach we believe is crucial if CCE is to be scaled sustainably. For comparison, ESGE sets a ≥ 90 % cecal intubation rate for colonoscopy, with a target rate ≥ 95 % 6 . Only CareForColon2015 met this benchmark, suggesting that current real-world CCE implementations still face challenges in reaching completeness rates on par with colonoscopy. Supplementary Table 2 is an overview of complete test definitions.

## Follow-up endoscopy

CCE lacks therapeutic capabilities and must be followed by a colonoscopy if indicated, i.e., by significant pathology or an incomplete investigation. NHS England (46,7%) reported a rate of follow-up endoscopy (FUE) that is notably lower than in the other studies, indicating effective patient selection 1 . Minimizing need for FUE or further testing is essential for CCE to be both a clinically viable and economically sustainable diagnostic option. Despite achieving a 69.2% success rate, CareForColon2015 reported a 69.9% FUE rate, highlighting that CCE is not a panacea for FIT-positive screening patients 2 . Due to the very high rate of FUE, CCE cannot be recommended as a standalone tool in population-wide colorectal screening. Instead, effective patient selection should be implemented, prioritizing patient groups with a low rate of positive findings 2 .

## Colorectal cancer

In the context of clinical implementation of CCE, concerns persist regarding its sensitivity in detecting colorectal cancers (CRCs) and advanced adenomas 2 . For CCE to be a viable alternative, its sensitivity must match or exceed that of colonoscopy. In NHS England, no cases of CRC were missed in complete and adequately prepared procedures. However, 14 cases of missed CRC occurred in patients with either incomplete CCE (where the cancer segment was not visualized) or inadequate bowel preparation, highlighting the importance of colonic cleanliness 1 . Neither of the ScotCap studies reported missed CRC 3 4 ; however, following publication of the ScotCap pilot, one case of missed CRC at the cecal pole was subsequently identified and published in a case report 11 . All figures are reported to the best of our knowledge and emphasize the critical role of test quality, reader vigilance, and specific attention to anatomic “areas of concern” for CCE, such as the cecal pole 11 . An overview of missed CRC cases can be found in Supplementary Table 3.

## Polyp detection rate

Sensitivity of CCE for detecting polyps ≥ 10 mm in the NHS England and ScotCap trials ranges from 93.8% to 97.0% on a per-patient basis, and from 75.0% to 95.8% on a per-polyp basis, when subsequent colonoscopy is used as the reference standard (Supplementary Table 4). NHS England reported the highest per-patient sensitivity (97%) for polyps ≥ 6 mm and ≥ 10 mm, but had the lowest per-polyp sensitivity, at 79.0% and 75.0%, respectively 1 . In contrast, the ScotCap pilot demonstrated lower per-patient sensitivity at 89.9% for ≥ 6 mm and 93.8% for ≥ 10 mm, but slightly higher per-polyp sensitivities of 91.0% and 95.2%, respectively 3 . The ScotCap registry achieved the highest per-polyp sensitivities at 97.1% for ≥ 6 mm and 95.8% for ≥ 10 mm, and per-patient sensitivities of 95.2% and 94.9%, respectively 4 . Turvill et al. suggest that lower sensitivity on a per-polyp basis is likely attributable to difficulties in accurately localizing the polyp within the colon 1 . Inaccurate localization can result in a true positive being misclassified as both a false negative and a false positive when findings are matched to those from the subsequent colonoscopy.

Across studies, CCE consistently reports a higher number of “false-positive” polyps compared with findings from follow-up colonoscopy 1 3 4 . Although this may partly be attributable to potential double-reporting by CCE readers, it is also highly plausible that CCE detects additional true polyps not detected during colonoscopy. For instance, Turvill et al. reported that more polyps were detected in the CCE arm than in the colonoscopy arm, both on a per-patient and per-polyp basis 1 . Similarly, the CareForColon2015 trial observed a higher rate of detection of CRCs and advanced adenomas in the CCE arm compared with the colonoscopy arm, although this difference did not reach statistical significance 2 .

A meta-analysis by Sulbaran et al. reported that in seven studies, so-called "false-positive" polyps identified by CCE prompted an additional unblinded colonoscopy, during which these polyps were in fact confirmed. This indicates that the lesions had been missed during the initial colonoscopy preformed after the CCE procedure. These findings suggest that, in certain contexts, the polyp detection rate of CCE may exceed that of colonoscopy 12 .

## Conclusions

In summary, these comparative data across national CCE programs highlight the inherent complexity of implementing CCE at scale. Variations in definitions, bowel preparation protocols, and center-level logistics underscore need for harmonized quality metrics. We believe it is essential to establish shared standards for “success,” prioritize reduction of downstream procedures, and, ideally, create platforms for shared learning between systems. The work by Turvill et al. sets a new benchmark in England, demonstrating what is possible with structured implementation and ongoing optimization. We suggest that future evaluations incorporate preprocedure, patient-level stratification (e.g., FIT values, symptom clusters) and technical audit loops to better understand variability in outcomes across centers. We commend the authors for their contribution to the evidence base and look forward to further dialogue as national CCE strategies evolve.

## Supplementary Material

Supplementary Material Zusatzmaterial
