# Supplementary material for: Colon capsule endoscopy today: Brief overview of leading UK and Danish initiatives
Source: Endosc Int Open. 2025 Jul 23;13:a26415952. doi: 10.1055/a-2641-5952 (PMC12303154; doi:10.1055/a-2641-5952)
Supplement: Supplementary file 1 — Supplementary Material [file 10-1055-a-2641-5952_26501820.pdf]

Supplementary Table 1 Definition of adequate bowel preparation.

NHS England	Score ≥ 6 on Colon Capsule Clear Score (CC-Clear)
ScotCap pilot	Adequate if rated ≥ “fair” in all colonic segments and deemed adequate overall by CCE reader per the Boston Bowel Preparation Scale
ScotCap registry	Adequate if rated ≥ “fair” in all colonic segments and deemed adequate overall by CCE reader per the Leighton–Rex bowel preparation scale
CareForColon2015	Adequate if rated ≥ fair on the Leighton–Rex scale for all colonic segments

Endosc Int Open | DOI 10.1055/a-2641-5952 | © The Author(s).

Supplementary Table 2 Definition of complete test.

NHS England	Capsule seen expelled or anal cushions identified
ScotCap pilot	Excretion of the capsule within its battery life or visualization of anal cushions
ScotCap registry	Visualization of whole colon and rectum
CareForColon2015 [10]	Visualization of hemorrhoidal plexus

Supplementary Table 3 Reported missed CRC at CCE.

NHS England	No CRCs were missed in complete and adequately prepared procedures<br>14 CRCs were missed due to either incomplete CCE (i.e. tumour-containing segments not visualized) or inadequate bowel preparation<br>One interval CRC was reported, but deemed to have occurred after both CCE and follow-up colonoscopy
ScotCap pilot	No CRCs were detected by CCE or follow-up testing; however, one subsequent cecal CRC was later reported in a separate case report
ScotCap registry	No missed CRC reported
CareForColon2015	Data not published yet

CCE, colon capsule endoscopy; CRC, colorectal cancer.

Supplementary Table 4 Sensitivity of CCE for detecting polyps ≥ 10 mm.

Study	Sensitivity of CCE per-patient	Sensitivity of CCE per-polyp
NHS England	97.0% (≥ 6 mm)<br>97.0% (≥ 10 mm)	79.0% (≥ 6 mm)<br>75.0% (≥ 10 mm)
ScotCap pilot	89.9% (≥ 6 mm)<br>93.8% (≥ 10 mm)	91.0% (≥ 6 mm)<br>95.2% (≥ 10 mm)
ScotCap registry	95.2% (≥ 6 mm)<br>94.9% (≥ 10 mm)	97.1% (≥ 6 mm)<br>95.8% (≥ 10 mm)
CareForColon2015	-	-

CCE, colon capsule endoscopy.

Endosc Int Open | DOI 10.1055/a-2641-5952 | © The Author(s).
